# Sodium-calcium exchanger and multiple sodium channel isoforms in intra-epidermal nerve terminals

**DOI:** 10.1186/1744-8069-6-84

**Published:** 2010-11-30

**Authors:** Anna-Karin Persson, Joel A Black, Andreas Gasser, Xiaoyang Cheng, Tanya Z Fischer, Stephen G Waxman

**Affiliations:** 1 Department of Neurology and Center for Neuroscience and Regeneration Research, Yale University School of Medicine, New Haven, CT 06510, USA; 2 Rehabilitation Research Center, Veterans Affairs Connecticut Healthcare System, West Haven, CT 06516, USA

## Abstract

**Background:**

Nociception requires transduction and impulse electrogenesis in nerve fibers which innervate the body surface, including the skin. However, the molecular substrates for transduction and action potential initiation in nociceptors are incompletely understood. In this study, we examined the expression and distribution of Na^+^/Ca^2+ ^exchanger (NCX) and voltage-gated sodium channel isoforms in intra-epidermal free nerve terminals.

**Results:**

Small diameter DRG neurons exhibited robust NCX2, but not NCX1 or NCX3 immunolabeling, and virtually all PGP 9.5-positive intra-epidermal free nerve terminals displayed NCX2 immunoreactivity. Sodium channel Na_V_1.1 was not detectable in free nerve endings. In contrast, the majority of nerve terminals displayed detectable levels of expression of Na_V_1.6, Na_V_1.7, Na_V_1.8 and Na_V_1.9. Sodium channel immunoreactivity in the free nerve endings extended from the dermal boundary to the terminal tip. A similar pattern of NCX and sodium channel immunolabeling was observed in DRG neurons *in vitro*.

**Conclusions:**

NCX2, as well as Na_V_1.6, Na_V_1.7, Na_V_1.8 and Na_V_1.9, are present in most intra-epidermal free nerve endings. The presence of NCX2, together with multiple sodium channel isoforms, in free nerve endings may have important functional implications.

## Background

Nociception begins with transduction and action potential electrogenesis in nerve fibers, many of which terminate close to the body surface in the skin epidermis. However, our knowledge of the molecular makeup of peripheral epidermal nerve terminals, which include nociceptors, remains incomplete. Distinct populations of free nerve terminals express CGRP or Mrgprd and have been shown to have P2Y2, P2X3 and TRPV1 receptors which are known to contribute to transduction by free nerve endings [1-4]. However, while it is now well-recognized that dorsal root ganglion (DRG) neurons, including nociceptors, express multiple sodium channel isoforms which contribute to electrogenesis within these cells [5,6], the regional distribution of these channel isoforms within nociceptor cell bodies, axons, and free nerve terminals is less well understood, in part as a result of the small (< 0.5 μm) diameters of the epidermal nerve endings. Most functional studies on sodium channel expression of DRG neurons have focused on the cell bodies which are amenable to patch clamp recording. In contrast, only a few electrophysiological studies have examined the contribution of sodium channels to the function of sensory terminals [7-10]. Although these studies provide evidence for the presence of tetrodotoxin (TTX)-sensitive and TTX-resistant sodium channel subtypes in nociceptive nerve terminals, most of these studies examined corneal [7,8] and epidural [9] nerve endings and only one study [10] focused on axons innervating the skin, and these studies do not provide a comprehensive demonstration of the pattern of expression of the sodium channel isoforms within the distal terminal endings of nociceptive afferents.

The Na^+^/Ca^2+ ^exchanger (NCX) normally operates as an antiporter that extrudes Ca^2+ ^from the cell. Three distinct isoforms of NCX (NCX1-3) have been cloned [11-13], and all are expressed in the CNS [14]. NCX has been linked to functions that include vesicle recycling and pre-synaptic excitability in the terminals of cultured neurons [15]. In the PNS, NCX is known to be expressed [16] and functional [17] along at least some myelinated axonal trunks, and has been shown to be present within cell bodies of DRG neurons, where it appears to play a role in Ca^2+ ^homeostasis [18]. However, it is not known whether NCX is present along non-myelinated axons, and expression of NCX in intra-epidermal free nerve terminals has not been studied.

In this paper, we demonstrate the presence of the NCX2 isoform of the Na^+^/Ca^2+ ^exchanger within free nerve endings within the epidermis. In addition, we demonstrate the expression of sodium channels Na_V_1.6, Na_V_1.7, Na_V_1.8 and Na_V_1.9 within axons composing small nerve bundles subjacent to epidermis and in the epidermal free nerve terminals. NCX2 and these sodium channel isoforms are present within the free nerve endings from the dermal boundary to the tips of the slender intra-epidermal endings. The presence of NCX2, together with sodium channel isoforms Na_V_1.6, Na_V_1.7, Na_V_1.8, and Na_V_1.9, may have important implications for the function and pathophysiology of small-diameter sensory nerve endings.

## Results

**Figure 1:**
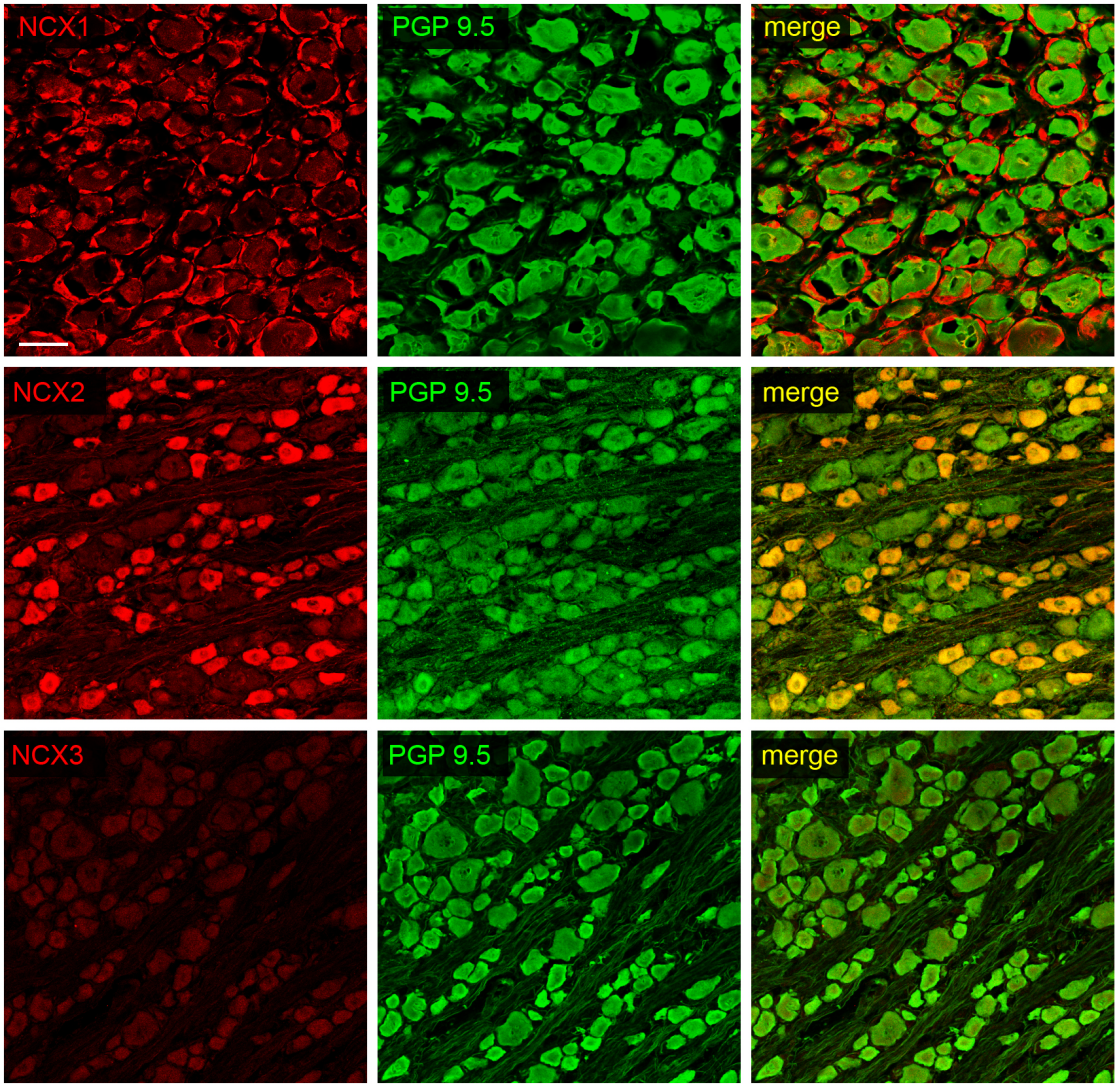
**Na^+^/Ca^2+ ^exchanger isoforms in rat dorsal root ganglion**. NCX1 is not detectable in DRG neurons (PGP 9.5 positive), but exhibits robust expression in the ensheathing satellite cells. NCX2 is highly expressed in DRG neurons with small diameters. Low levels of NCX3 are present in most neurons irrespective of cell body diameter. NCX1, 2, and 3: red; PGP 9.5: green; merge: yellow. Scale bar, 50 μm.

In this study, we investigated the expression and distribution of Na^+^/Ca^2+ ^exchanger (NCX) and voltage-dependent Na^+ ^channel isoforms in axons and free nerve terminals within skin dermis and epidermis. To establish the identity of NCX isoforms that are expressed by small diameter (< 25 μm) dorsal root ganglia (DRG) neurons, which give rise to C- and Aδ-fibers that terminate as free nerve endings in the skin, we reacted sections of DRG with antibodies specific to NCX isoforms. As shown in Figure 1 DRG neurons of all size classes exhibited minimal NCX1 immunolabeling. However, satellite cells ensheathing DRG neurons displayed robust NCX1 reactivity. In contrast to the limited NCX1 labeling in all DRG neurons, NCX2 immunolabeling was prominent in small diameter DRG neurons and was generally not above background levels in larger diameter neurons (Figure 1). Virtually all small diameter DRG neurons exhibited NCX2 immunolabeling. DRG neurons of all size classes displayed a low level of NCX3 labeling.

**Figure 2:**
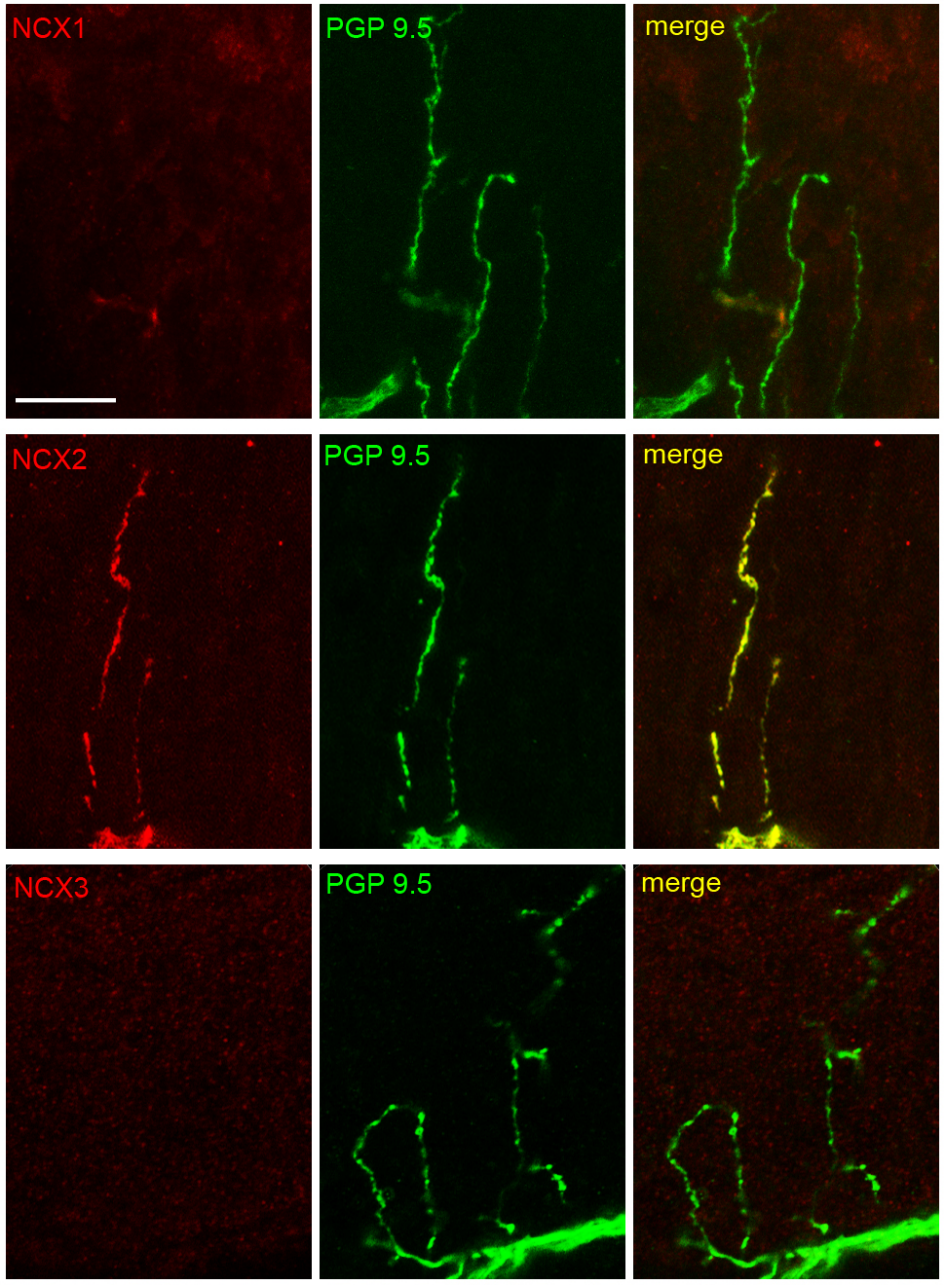
**NCX expression in free nerve endings within epidermis**. PGP 9.5-positive free nerve endings exhibit NCX2, but not NCX1 and NCX3 immunolabelling. NCX2 labeling extends from the dermal boundary to the tip of the nerve terminal. NCX: red; PGP 9.5: green; merge: yellow. Scale bar, 10 μm.

**Figure 3:**
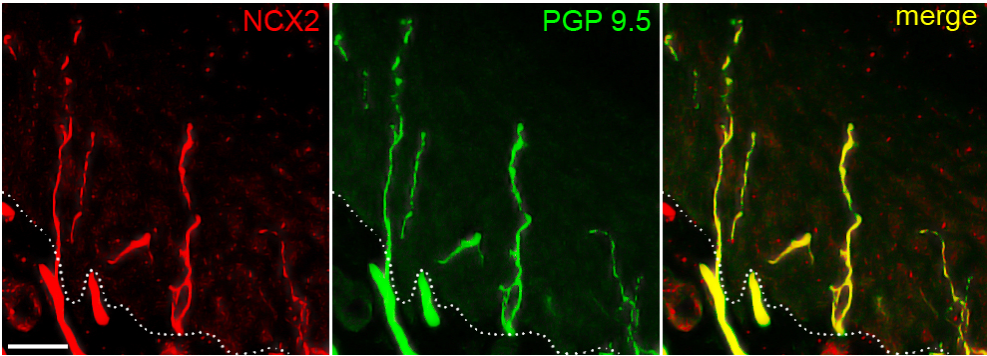
**NCX2 expression in dermal nerve bundles and epidermal nerve endings**. Small bundles of axons in the dermis subjacent to the epidermis exhibit NCX2 immunolabeling. Virtually all PGP 9.5-positive free nerve endings in the epidermis display NCX2 immunoreactivity. Dashed line indicates the border between epidermis (top) and dermis (bottom). NCX2: red; PGP 9.5: green; merge: yellow. Scale bar, 20 μm.

Free nerve terminals in the epidermis, in most cases with diameters of less that 0.5 μm, were identified by labeling with an antibody to PGP 9.5. In epidermis, NCX1 was not detectable in free nerve endings (Figure 2). In contrast, PGP 9.5-positive free nerve terminals displayed robust immunolabeling for NCX2, consistent with expression of NCX2 in the cell bodies of small diameter DRG neurons. NCX3 immunolabeling was not detectable in free nerve endings in epidermis, although we were able to discriminate low levels of NCX3 reactivity in small diameter DRG neurons. In sections of epidermis that displayed multiple free nerve terminals (Figure 3), NCX2 immunolabeling was exhibited by all PGP9.5-positive nerve endings. We assessed the labeling of 50 PGP 9.5-positive free nerve endings within skin samples and detected NCX2 labeling in all nerve endings. Prominent NCX2 immunoreactivity was also displayed by the small nerve bundles of axons within the dermis immediately subjacent to the epidermis (Figure 3).

**Figure 4:**
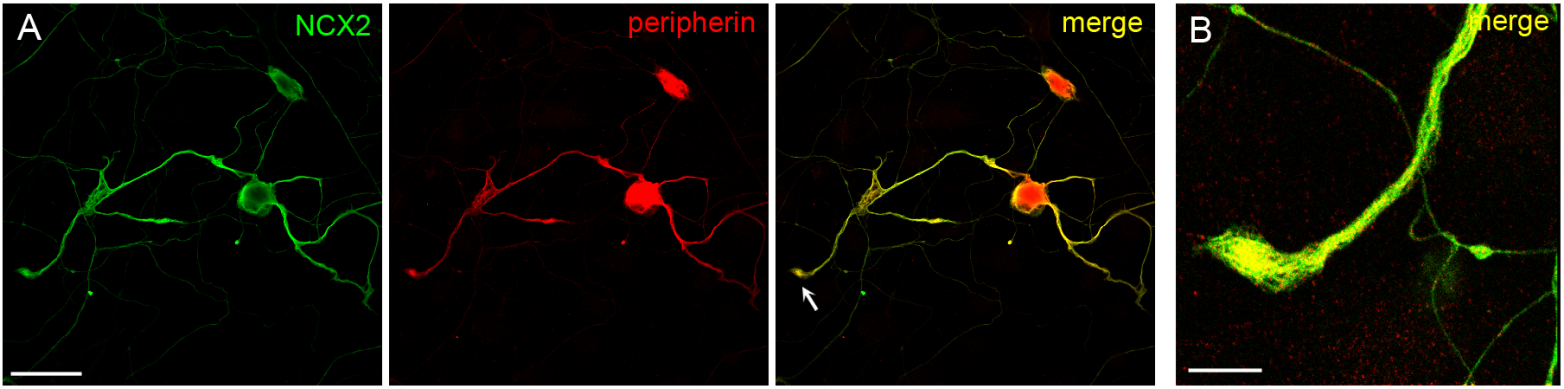
**NCX2 expression in DRG neurons *in vitro***. **A**. NCX2 (green) immunoreactivity in the cell body and neurites of a peripherin-positive (red) neuron. Merged image (yellow) demonstrates co-localization of NCX2 and peripherin. **B**. High magnification image showing NCX2 (yellow = merge of NCX2 and PGP 9.5 signals) present at a neurite tip (indicated with arrow, left image). Scale bars, 50 μm (left); 10 μm (right).

We also examined cultured DRG neurons to determine whether the DRG neuronal cell bodies and processes *in vitro *displayed a similar pattern of NCX labeling as observed *in vivo*. Consistent with the labeling pattern observed *in vivo*, NCX1 immunolabeling was not detectable and only extremely low levels of NCX3 reactivity were exhibited in cultured neuronal cell bodies and neurites (data not shown). In contrast, cultured DRG neurons exhibited robust NCX2 immunoreactivity within cell bodies and neurites (Figure 4A). The NCX2 labeling of neurites extended throughout their entire length, and was clearly present at the neuritic tips (Figure 4B).

**Figure 5:**
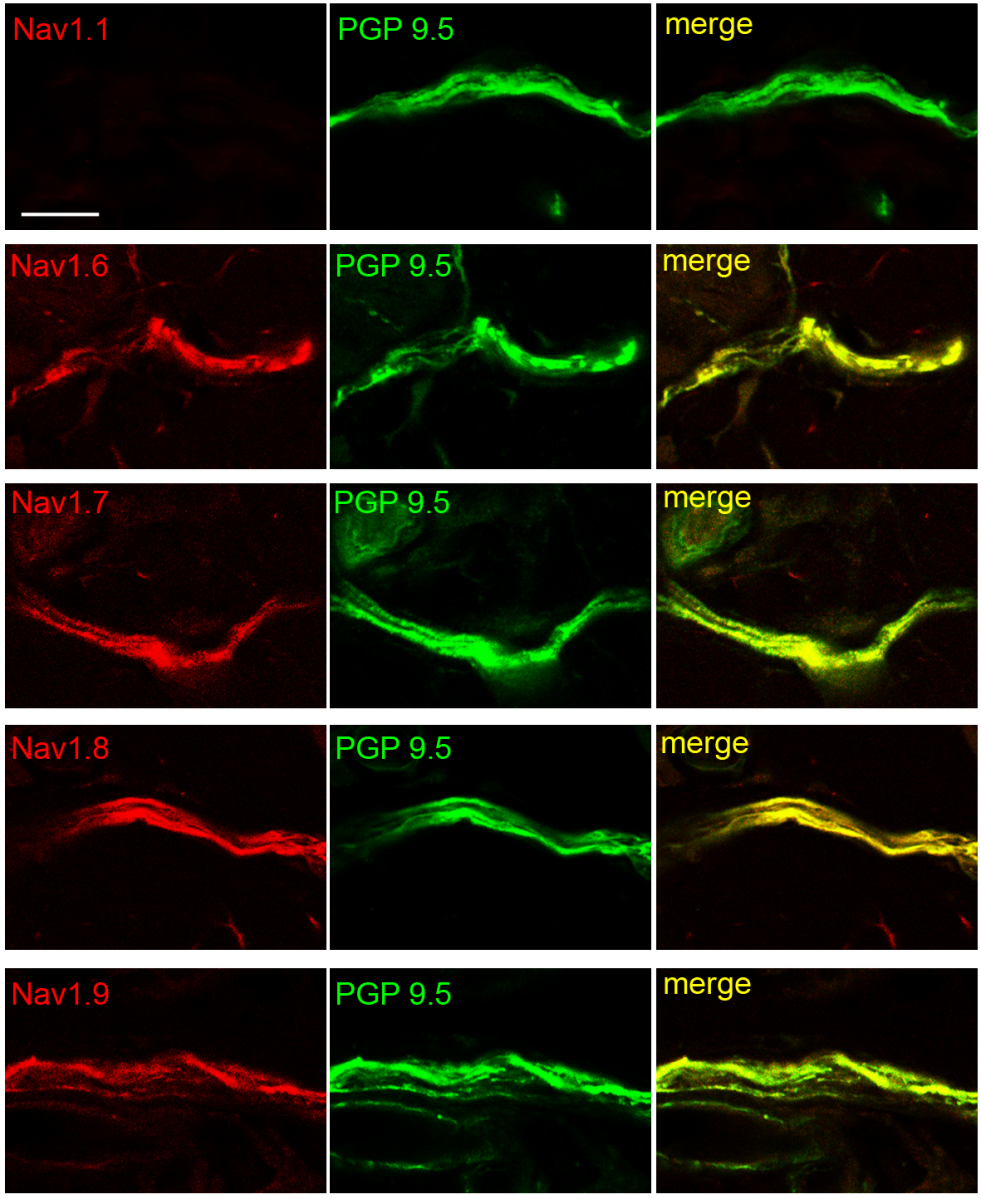
**Sodium channel expression in nerve bundles within the dermis**. The small bundles of fibers within the dermis subjacent to the epidermis exhibit Na_V_1.6, Na_V_1.7, Na_V_1.8 and Na_V_1.9 immunolabeling. Na_V_1.1 immunolabeling is not detectable within the small nerve bundles. Sodium channels: red; PGP 9.5: green; merge: yellow. Scale bar, 10 μm.

To examine the expression of sodium channels within axons and free nerve terminals of skin dermis and epidermis, we reacted sections of skin tissue with antibodies specific for sodium channel isoforms that are expressed within DRG neurons (i.e. Na_V_1.1, Na_V_1.6, Na_V_1.7, Na_V_1.8 and Na_V_1.9; [19]). In the small nerve bundles of PGP 9.5-positive fibers within dermis immediately subjacent to epidermis, Na_V_1.1 labeling was not detectable (Figure 5), consistent with limited Na_V_1.1 expression that has been reported within small DRG neurons [20]. In contrast, robust labeling for Na_V_1.6, Na_V_1.7, Na_V_1.8 and Na_V_1.9 was exhibited in the PGP 9.5-positive axons in the small nerve bundles within the dermis. The labeling for these sodium channels was generally evenly distributed along the axons and was not patchy or aggregated in specific regions of the PGP 9.5-positive fibers.

**Figure 6:**
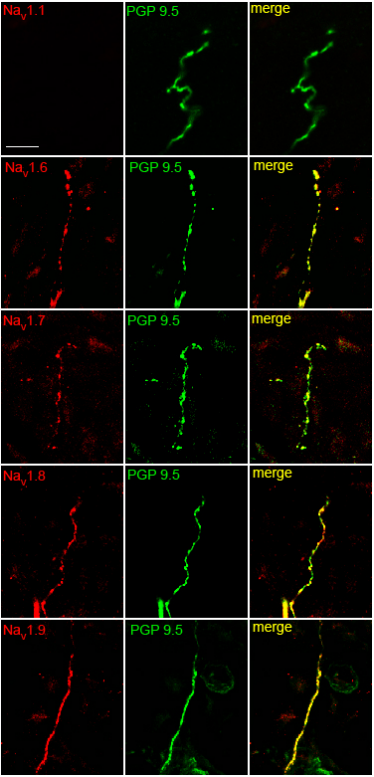
**Sodium channel expression within free nerve endings in epidermis**. PGP 9.5-positive (green) free nerve endings exhibit Na_V_1.6, Na_V_1.7, Na_V_1,8 and Na_V_1.9 immunolabeling (red). Na_V_1.1 is not detectable in epidermal nerve fibers. Scale bar, 10 μm.

Axons that exit the nerve bundles become fine caliber (~0.2 - 0.5 μm diam.) free nerve endings upon passing through the basement membrane separating dermis from epidermis; these nerve terminals extend towards the skin surface with a tortuous, non-linear trajectory and often exhibit varicosities of variable sizes along their lengths, further complicating analysis. Nonetheless, in favorable sections we observed a pattern of sodium channel immunolabeling that paralleled that in DRG neuronal cell bodies. As anticipated, Na_V_1.1 immunolabeling was not detectable in the PGP 9.5-positive free nerve endings (Figure 6). However, Na_V_1.6, Na_V_1.7, Na_V_1.8 and Na_V_1.9 immunolabeling was clearly displayed by PGP 9.5-positive free nerve terminals. While difficult to assess due to the small diameter and tortuous course exhibited by free nerve terminals in epidermis, the sodium channel immunoreactivity appeared to extend to the tips of the endings, being co-localized with PGP 9.5 along the length of the nerve terminals to the distal tips. The varicosities of the free nerve endings exhibited clear labeling for these channels, with generally less intense staining in the regions between the varicosities. The percentage of PGP 9.5-positive nerve terminals that exhibited Na_V_1.6, Na_V_1.7, Na_V_1.8 and Na_V_1.9 immunolabeling in skin was assessed in the most heavily stained sections (n = 50 nerve endings per sodium channel isoform). Na_V_1.6, Na_V_1.7, Na_V_1.8 and Na_V_1.9 immunolabeling was detectable in 70%, 90%, 96% and 93%, respectively, of PGP 9.5-positive nerve terminals.

**Figure 7:**
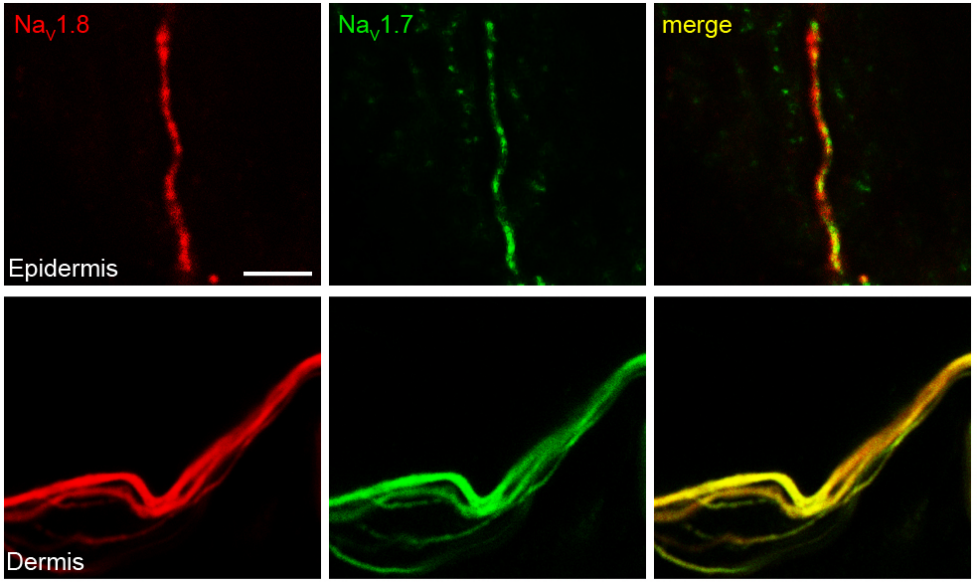
**Co-localization of Na_V_1.8 and Na_V_1.7 in epidermal nerve endings**. Na_V_1.8 (red) and Na_V_1.7 (green) are co-localized (yellow) in dermal nerve bundles subadjacent to the epidermis and in single intra-epidermal nerve terminals. Scale bar, 5 μm.

Since Na_V_1.7 and Na_V_1.8 are physiologically coupled and the presence or absence of co-expression of Na_V_1.7 and Na_V_1.8 can markedly alter the excitability of neurons [21], we also used double label immunostaining to directly examine whether these sodium channel isoforms are co-expressed in free nerve terminals. As shown in Figure 7 Na_V_1.7 and Na_V_1.8 were co-localized in axons within small nerve bundles adjacent to the epidermis and free nerve endings within the epidermis. Na_V_1.7 and Na_V_1.8 were both present and were co-localized within the varicosities and inter-connecting regions of the nerve terminals.

**Figure 8:**
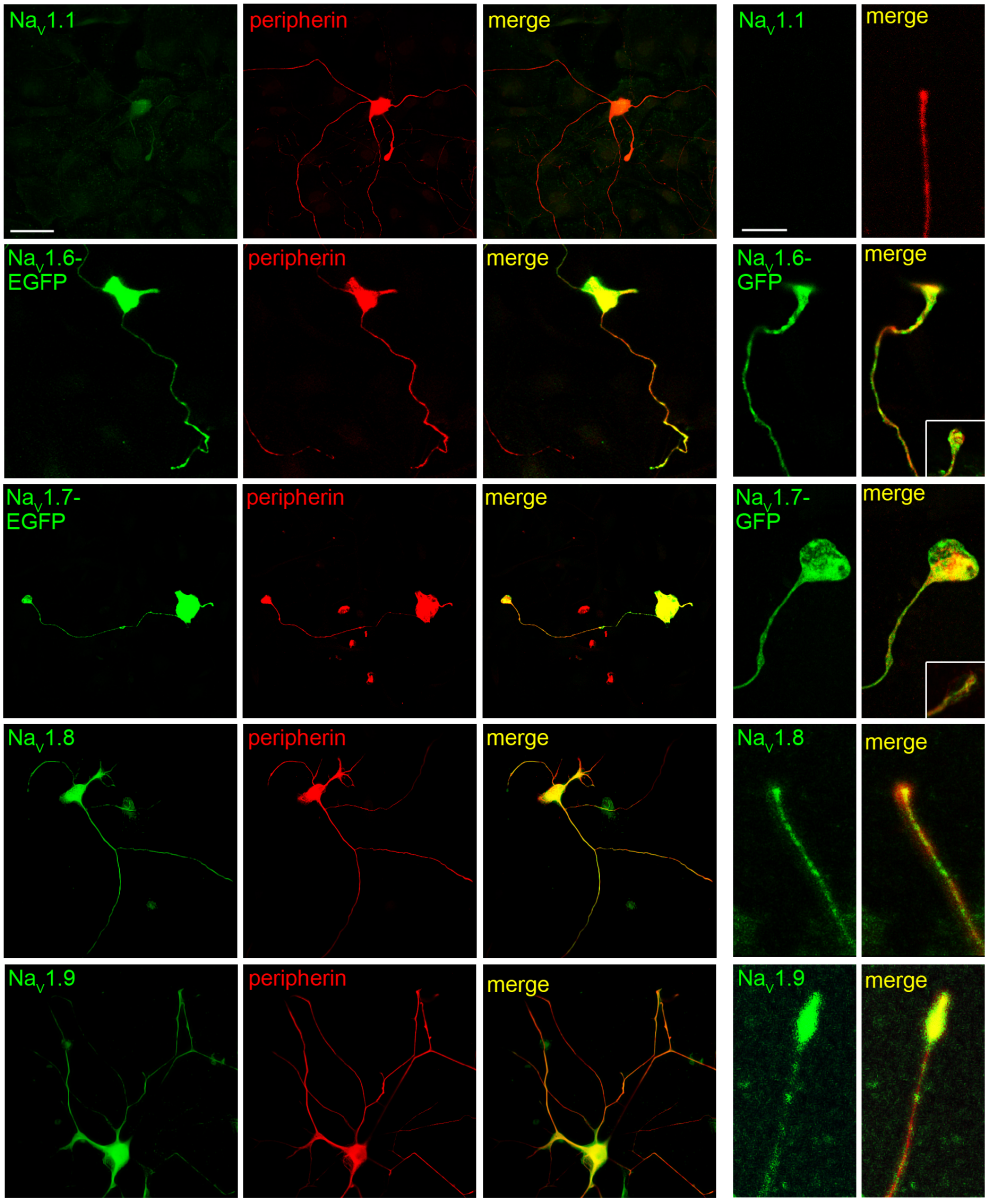
**Sodium channel expression in DRG neurons *in vitro***. Left panels: Na_V_1.6-EGFP, Na_V_1.7-EGFP, Na_V_1,8 and Na_V_1.9 immunolabeling is displayed within DRG cell bodies and neurites. Na_V_1.1 is detectable at low levels in the cell body and only in neurites close to the cell body. Right panel: High magnification of neurite endings: tips of free nerve terminals exhibit Na_V_1.6-EGFP, Na_V_1.7-EGFP, Na_V_1.8 and Na_V_1.9 immunolabeling. Na_V_1.1 immunostaining is not detectable in neuritic nerve ending. Sodium channels: green; PGP 9.5: red; merge: yellow. *Insets*: Peripherin-positive (green) neurite tips exhibit immunolabeling (red) with antibodies directed against Na_V_1.6 and Na_V_1.7. Scale bar, 50 μm (left), 10 μm (right).

As with the distribution of NCX isoforms in cultured DRG neurons, we determined whether sodium channels Na_V_1.1, Na_V_1.6, Na_V_1.7, Na_V_1.8 and Na_V_1.9 are targeted to neurites and are translocated to neuritic tips in DRG neurons *in vitro*. For this examination, the distribution of Na_V_1.1, Na_V_1.8 and Na_V_1.9 in neurites was assessed with isoform-specific antibodies to these channels. For Na_V_1.6 and Na_V_1.7, we also employed transfection of DRG neurons with Na_V_1.6-EGFP and Na_V_1.7-EGFP constructs, supplemented with anti-GFP antibody detection. While Na_V_1.1 is expressed within cell bodies of some DRG neurons *in vitro*, Na_V_1.1 immunolabeling was detected only in the most proximal peripherin-positive neurites and was not apparent at the neuritic tips (Figure 8). In contrast, Na_V_1.6, Na_V_1.7, Na_V_1.8 and Na_V_1.9 sodium channels are distributed along the lengths of peripherin-positive neurites, which often displayed multiple branching and extensive lengths. Importantly, the distribution of the sodium channels extended to the neuritic endings (Figure 8 rightmost panels), with many neurite tips exhibiting extensive aggregation of the channels. Non-transfected cultures of DRG neurons, immunolabeled with isoforms-specific Na_V_1.6 and Na_V_1.7 antibodies, displayed similar patterns of sodium channel expression along the neurites as the transfected neurons (Figure 8 insets).

## Discussion

In this study, we have demonstrated the presence of the NCX2 isoform of the Na^+^/Ca^2+ ^exchanger (NCX), together with sodium channel isoforms Na_V_1.6, Na_V_1.7, Na_V_1.8, and Na_V_1.9 within intra-epidermal free nerve terminals which include nociceptive endings. Our results are novel in demonstrating the presence of NCX2 in virtually all of the small-diameter axons and their terminals in the epidermis, and show that Na_V_1.6, Na_V_1.7, Na_V_1.8, and Na_V_1.9 are detectable in a majority of epidermal nerve endings. Consistent with our observation of NCX2 in these terminals, Verdru et al. [18] reported sodium-calcium exchange, and expression of mRNAs for multiple NCX isoforms including NCX2, in rat DRG neurons, and our studies demonstrate NCX2 within DRG neuronal cell bodies. Our observations in epidermal nerve endings extend earlier observations by Toledo-Aral et al. [22] who observed Na_V_1.7 within the growth cones of DRG neurons in culture, Black et al. [23] who observed Na_V_1.6 in axons within the skin, and Zhao et al. [24] who described Na_V_1.8 and Na_V_1.9 in nerve endings in epidermis. Notably, our results demonstrate that NCX2 is present within virtually all epidermal nerve terminals and thus indicate that NCX is present in terminals that express Na_V_1.6, Na_V_1.7, Na_V_1.8 and Na_V_1.9. Our results also demonstrate the co-localization of Na_V_1.7 and Na_V_1.8 within intra-epidermal nerve fibers, a result that is functionally important since Na_V_1.7 and Na_V_1.8 play different and interdependent roles in electrogenesis [6,21].

Electrophysiological recordings indicate that a tetrodotoxin (TTX)-resistant sodium channel isoform, presumably Na_V_1.8 on the basis of kinetics, is expressed at levels that can support action potential electrogenesis, together with unspecified TTX-sensitive sodium channels that contribute to excitability, within terminal branches of C- and Aδ-type dural afferent axons [9]. Similarly, TTX-resistant sodium channels have been shown to support action potentials within polymodal, mechanosensory, and cold-sensitive nerve terminals in the cornea, where action potential electrogenesis is also partially supported by TTX-sensitive sodium channels [7]. There is also evidence indicating that TTX-resistant sodium channels (probably Na_V_1.8) allow the superficial endings of slowly conducting nociceptive fibers to conduct impulses at low temperatures [10]. A role of sodium channels in transduction in sensory axon terminals is also suggested by the observation that 6.0 μM TTX attenuates receptor potentials in Pacinian corpuscles [25], although this concentration does not differentiate between TTX-sensitive and TTX-resistant channels and thus provides no information about the molecular identity of the channels. There is also electrophysiological evidence indicating that, along C-fiber trunks, Na_V_1.6 [23] and sodium channels with physiological properties suggestive of Na_V_1.7 and Na_V_1.8 [26] contribute to action potential propagation.

It is possible that some intra-epidermal nerve fibers may express low densities of channels that are below the threshold for detection by immunocytochemical methods, so the percentages of fibers expressing sodium channel isoforms that we report may represent underestimates. Moreover, our results do not provide information about the density of functional sodium channels within the membrane of the epidermal nerve endings. Low sodium channel densities (as low as 1-3/μm^2^) can support action potential electrogenesis in neuronal compartments such as small-diameter axons with high input impedances [27,28]. There may in fact be functional benefit to limiting sodium channel density within sensory terminals since even moderate sodium channel densities (30/μm^2 ^in a 1.0 μm diameter axon) can produce spontaneous firing as a result of channel noise or spontaneous channel opening in the context of a high input resistance, low capacitance per unit length, and shorter length constant [29-31].

While the presence of sodium channels along axons is not unexpected, the precise functional roles of each of the four sodium channel isoforms within intra-epidermal nerve terminals remain to be determined. The sodium channel isoforms that we have observed in intra-epidermal nerve terminals display a spectrum of biophysical and physiological properties which tune them so that they support sodium influx over a range of voltage and time domains. Na_V_1.6, the major channel at nodes of Ranvier [32], is also present along the trunks of central non-myelinated axons [33] and peripheral C-fibers [23] and, as a result of rapid recovery from inactivation [34], produces sodium influx that contributes to high-frequency firing. Na_V_1.7 displays slow closed-state inactivation [34,35] and generates an inward sodium current in response to small slow depolarizing inputs such as generator potentials in the subthreshold range. Na_V_1.8 displays rapid recovery from inactivation [36] and depolarized activation and inactivation voltage-dependence [37] which permit it to generate a large inward sodium current during the rising phase of the action potential [38,39] including high-frequency firing in response to sustained depolarization [39]. Na_V_1.9 displays broad overlap between activation and inactivation together with extremely slow inactivation [40], and can generate a persistent sodium current at subthreshold potentials so as to amplify and prolong depolarizing inputs, decrease action potential threshold, and depolarize resting potential [41,42]. Although the majority of Na_V_1.9 [40] and possibly other sodium channels may be inactivated at the resting potential of sensory terminals [8], expression and current density of Na_V_1.7 [43,44], Na_V_1.8 [45-47] and Na_V_1.9 [6,41] are known to be up-regulated by pro-inflammatory molecules, so that these channels may contribute to sensory axon sensitization under conditions of inflammation.

Small caliber C- and Aδ-fibers that terminate as free nerve endings in the epidermis each represent spatially and molecularly heterogeneous populations, expressing non-uniform combinations of ligand-mediated ionotropic and metabotropic receptors, voltage-gated ion channels, heat/cold receptors and neuropeptides (see e.g. [1]). For instance, nerve endings expressing the G-protein coupled receptor Mrgprd terminate in the stratum granulosum [4], where they are suggested to play an important role in noxious mechanical nociception [48]. Conversely, the non-overlapping peptidergic, TRPV1^+ ^nerve endings terminate in stratum spinosum and appear to participate in noxious heat sensitivity [4,48]. Most intra-cutaneous nerve terminals express Na_V_1.7, Na_V_1.8 and Na_V_1.9, while Na_V_1.6 is detectable in 70% of the nerve endings. It is not clear whether the different subsets of free nerve endings exhibit different patterns of sodium channel expression.

The expression of NCX2 along intra-epidermal nerve terminals provides a molecular substrate for sodium-calcium exchange in these fibers. NCX is known to be present along the trunks of myelinated axons [16] where, under normal (non-pathological) conditions, sodium-calcium exchange is coupled to sodium influx, and contributes to calcium extrusion following physiological activity [17]. However, the presence of NCX2 together with sodium channels within epidermal nociceptive terminals may also have pathophysiological implications. Even a small ongoing sodium influx may increase intracellular sodium levels within small-diameter axons, which have a large surface-volume ratio, thereby imposing a substantial energetic load [49]. Persistent currents and ramp responses to small, slow depolarizations are produced over multiple overlapping voltage domains extending from the resting potential of DRG neuron somata to nearly 0 mV by the sodium channels present within these sensory terminals, Na_V_1.6 [50,51], Na_V_1.7 [35], Na_V_1.8 [52] and Na_V_1.9 [40]. Persistent sodium influx via sodium channels has been shown to drive injurious, calcium-importing reverse sodium-calcium exchange in myelinated axons under conditions of energy deprivation such as anoxia or ischemia [53-55]. In addition, repetitive action potential activity at physiological frequencies can also render axons vulnerable to metabolic insults so that a combination of energy deprivation and electrical activity can lead to axonal degeneration [56]. Expression of NCX2 together with sodium channels in intra-epidermal axon terminals may thus make these fine-diameter nerve fibers especially sensitive to injury when energetically challenged.

## Conclusions

In this study we examined the expression and distribution of isoforms of the sodium-calcium exchanger (NCX) and voltage-gated sodium channels Na_V_1.6, Na_V_1.7, Na_V_1.8 and Na_V_1.9 in intra-epidermal free nerve endings, which include nociceptors. Our data demonstrate that the sodium-calcium exchanger isoform NCX2 is expressed in nearly all intra-epidermal nerve endings. We also demonstrate that most intra-epidermal nerve endings express sodium channels Na_V_1.6, Na_V_1.7, Na_V_1.8 and Na_V_1.9. Similar patterns of sodium channel and NCX2 expression were demonstrated along the neurites and at the neuritic tips of cultured DRG neurons. The expression of NCX2 and sodium channels may have implications for the physiology and pathophysiology of fine-diameter intra-epidermal nerve endings.

## Methods

### Animals

Male Sprague-Dawley rats (Harlan, Indianapolis, IN) were housed under a 12 hr light/dark cycle in a pathogen-free area with *ad libitum *access to water and food. Animal use followed guidelines established by NIH and a protocol approved by the VA Connecticut Healthcare System Institutional Animal Care and Use Committee.

### Antibodies

Antibodies against sodium channel isoforms Na_V_1.1 (rabbit; Alomone Laboratories, Jerusalem, Israel), Na_V_1.1 (mouse; Antibodies, Inc., Davis, CA), Na_V_1.6 (rabbit; Sigma-Aldrich Inc., St. Louis, MO), Na_V_1.7 (rabbit; Y083, [57]), Na_V_1.8 (rabbit; Alomone), Na_V_1.8 (mouse; Antibodies Inc) and Na_V_1.9 (rabbit; #6464, [58]) were used in this study. Na^+^/Ca^2+ ^exchanger (NCX) isoforms were detected using antibodies against, NCX1 (rabbit; Santa Cruz Biotechnology Inc., Santa Cruz, CA), NCX2 (goat; Santa Cruz Biotechnology Inc.) and NCX3 (rabbit; Lifespan Biosciences, Seattle, WA). Anti-Protein Gene Product (PGP) 9.5 (mouse; EnCor Biotechnology Inc., Gainesville, FL), anti-Peripherin (mouse; Abcam, Cambridge, MA) and anti-Peripherin (chicken; Aves Labs Inc., Tigard, OR) were used as neuronal markers and anti-GFP (rabbit; Invitrogen, Carlsbad, CA) to enhance EGFP signal of transfected neurons.

### Immunohistochemistry of intra-epidermal terminals

Adult (225-250 g) male rats were deeply anesthetized with ketamine/xylazine (80/8 mg/kg b.w., i.p.) and transcardially perfused with phosphate buffered saline (PBS) followed by a 4% paraformaldehyde solution in 0.14 M Sorensen's phosphate buffer. Dorsal root ganglia and the glabrous skin from the plantar surface of the hindpaws were dissected and cryoprotected with 30% sucrose in 0.14 M phosphate overnight at 4°C, cryostat sectioned (12 μm) and mounted on glass slides (Fischer SuperFrost Plus) prior to staining. Immunocytochemistry procedures were performed as previously described [58]. Briefly, sections were blocked with PBS containing 5% fish skin gelatin (Sigma), 3% normal donkey serum, 0.3% Triton X-100, and 0.02% sodium azide for 1 hour at room temperature. Subsequently, slides were incubated individually or in combination with primary antibodies (rabbit anti-Na_V_1.1, 1:100, rabbit anti-Na_V_1.6, 1:100, rabbit anti-Na_V_1.7, 1:200, rabbit anti-Na_V_1.8, 1:200, mouse anti-Na_V_1.8, 1:100, rabbit anti-Na_V_1.9, 1:500, rabbit anti-NCX1, 1:100, goat anti-NCX2, 1:100, rabbit anti-NCX3, 1:100, and mouse anti-PGP 9.5, 1:2000, diluted in blocking solution, for 2 days at 4°C. Following extensive PBS washes, tissue was incubated in appropriate secondary antibody(ies): donkey anti-mouse IgG-488 (1:1000, Invitrogen), donkey anti-mouse IgG-549 (1:750, Jackson ImmunoResearch, West Grove, PA), donkey anti-rabbit IgG-488, donkey anti-rabbit IgG-Cy3, donkey anti-goat 488 or donkey anti-goat 549 (all 1:500, Jackson ImmunoResearch) diluted in blocking solution for 1 day at 4°C. After extensive washes, slides were coverslipped with Aqua-poly-mount (Polysciences, Warrington, PA). Control experiments performed without primary antibodies did not show labeling above background levels (data not shown).

### Immunocytochemistry of DRG neurons in vitro

Cultured wildtype- or transfected rat DRG neurons were fixed with 4% paraformaldehyde for 10 minutes, washed, incubated with primary antibodies (rabbit Na_V_1.6, 1:100, rabbit Na_V_1.7, 1:200, mouse Na_V_1.8, 1:100, rabbit Na_V_1.9, 1:500, rabbit NCX1, 1:100, goat NCX2, 1:100, rabbit NCX3, 1:100, rabbit GFP, 1:1000, mouse peripherin, 1:1000, and chicken peripherin, 1:100, for 2 hours at room temperature, washed, incubated in donkey anti-mouse IgG-549 (1:750, Jackson ImmunoResearch), donkey anti-rabbit IgG-488, donkey anti-rabbit IgG-Cy3, donkey anti-goat IgG-488 or donkey anti-chicken 488 (all 1:500, Jackson ImmunoResearch) for 1 hour at room temperature. Following washing, neurons were mounted on glass slides with Aqua-poly-mount (Polysciences).

### Image acquisition and analysis

Tissue and coverslips were examined with a Nikon C1si confocal microscope (Nikon USA, Melville, NY). Using EZ-C1 software (Nikon USA), digital images were accrued from a single z plane, or as a series of digital images in the *x-y *plane incremented by 0.4-0.7 μm in the *z *plane. For assessment of the percentage of free nerve endings expressing NCX2 or sodium channel isoforms, PGP 9.5-positive free nerve endings were identified (blindly with respect to the sodium channel/NCX2 immunosignal) with the green channel and then the sodium channel or NCX2 labeling was visualized in the red channel. The percentages of nerve terminals exhibiting immunolabeling clearly above background were determined. Fields were chosen in which immunosignal from keratinocytes (which are known to express varying levels of a number of sodium channel isoforms [24]) was minimal, so as not to obscure the labeling of the much thinner axons and terminals. Images were processed and composed in Adobe Photoshop (Adobe Systems, Mountain View, CA).

### DRG cultures and transfection

Dorsal root ganglion neurons from (4-8 week old) rats were isolated and cultured as previously described [44], with neuronal enrichment as described by Shortland et al [59]. As an alternative method for visualization of Na_V_1.6 and Na_V_1.7, transfection of DRG neurons with pcDNA3-Na_V_1.6_R_-EGFP or pcDNA3-Na_V_1.7_R _-EGFP-vectors was performed as previously described [21]. Briefly, 3 μg of channel plasmid was electroporated into the freshly-isolated DRG neurons using Rat Neuron Nucleofector Solution (Lonza, Walkerville, MD). Following the plating of transfected DRG neurons onto laminin-coated glass coverslips, cultures were maintained in DMEM/F12 supplemented with 10% fetal calf serum, penicillin/streptomycin, and nerve growth factor and glial cell-derived neurotrophic factor (50 ng/ml each) at 37°C and 5% CO_2_. Images of transfected neurons were obtained 2-4 days following transfection.

### Plasmids

#### Na_V_1.6-EGFP

The plasmid pcDNA3-Na_V_1.6_R_, which encodes full-length mouse Na_V_1.6 rendered resistant to tetrodotoxin (TTX-R) by the Y371 S substitution [34], was modified by an in-frame fusion of enhanced green fluorescent protein (EGFP) to the C-terminus. By mutagenesis using QuikChange II XL mutagenesis kit (Stratagene, La Jolla, CA), a unique *KpnI *restriction site, which removed the translation termination codon (TAG), and a unique *XbaI *restriction site 3' of the *KpnI *site were introduced. The coding sequence for EGFP was then cut from pEGFP-N3 (BD Biosciences, San Jose, CA) with the same enzymes, and was cloned into the *KpnI *and *XbaI *sites of pcDNA3-Na_V_1.6 _R_. This resulted in the fusion of EGFP to the C-terminus of the Na_V_1.6 open reading frame with a linker sequence of 10 amino acids. Finally, the length of this linker was extended to 19 amino acids by insertion of oligonucleotides into the *KpnI *site, which improved the green fluorescence intensity of the fusion protein (data not shown). This final plasmid construct is referred to as Na_V_1.6-EGFP. ND7/23 cells were transiently transfected with the Na_V_1.6-EGFP vector and fast-activating and fast-inactivating TTX-R Na^+ ^currents comparable to Na_V_1.6_R _currents were recorded from ND7/23 cells exhibiting green fluorescence (unpublished data).

#### Na_V_1.7-EGFP

The pcDNA3-Na_V_1.7_R_-EGFP construct was generated by inserting an EGFP-coding sequence into pcDNA3-Na_V_1.7_R _using QuikChange II XL mutagenesis kit (Stratagene) and Quick ligation kit (NEB, Ipswich, MA). Briefly, the translation termination codon (TAG) of Na_V_1.7_R _was removed and two unique restriction sites (one *ApaI *and one *NotI *site) were introduced downstream the coding region of Na_V_1.7_R _using QuikChange II XL mutagenesis kit to generate pcDNA3-Na_V_1.7_R_-*ApaI-NotI *vector. The EGFP coding sequence was then amplified from pEGFP-N3 vector (BD Biosciences) with a forward primer containing an *ApaI *site and a reverse primer containing a *NotI *site. The EGFP fragments were digested with *ApaI *and *NotI *restriction endonucleases and cloned into pcDNA3-Na_V_1.7_R_-*ApaI-NotI *using T4 DNA ligase, generating pcDNA3-Na_V_1.7_R_-EGFP vector. The linker between the C-terminus of Na_V_1.7_R _and the N-terminus of EGFP is 16 amino acids in pcDNA3-Na_V_1.7_R_-EGFP vector. HEK293 cells were transiently transfected with pcDNA3-Na_V_1.7_R_-EGFP vector. Fast-activating and fast-inactivating Na^+ ^currents comparable to Na_V_1.7_R _currents were recorded from HEK293 cells exhibiting green fluorescence (unpublished data).

## Competing interests

The authors declare that they have no competing interests.

## Authors' contributions

Authors AKP and JAB collected, analyzed and interpreted immunohisto- and cytochemical data. AG designed and cloned Na_V_1.6-EGFP construct. XC designed and cloned Na_V_1.7-EGFP construct and participated in electrophysiological verification of clones. TZF participated in interpretation of immunocytochemical data. SGW conceived the project, participated in experimental design and interpretation. All authors participated in writing the manuscript and have read and approved the final version of the manuscript.
